# Bilateral primary adrenal lymphoma presenting with chylothorax: imaging findings and diagnostic challenges

**DOI:** 10.1093/bjrcr/uaag036

**Published:** 2026-08-18

**Authors:** Ubeyde Telli Oner, Nigar Erkoc, Musa Atay

**Affiliations:** Department of Radiology, University of Health Sciences, Bagcilar Training and Research Hospital-Istanbul, 34200, Türkiye; Department of Radiology, University of Health Sciences, Bagcilar Training and Research Hospital-Istanbul, 34200, Türkiye; Department of Radiology, University of Health Sciences, Bagcilar Training and Research Hospital-Istanbul, 34200, Türkiye

**Keywords:** primary adrenal lymphoma, diffuse large B-cell lymphoma, chylothorax, intratumoral hemorrhage, adrenal mass

## Abstract

Primary adrenal lymphoma is a rare and aggressive subtype of extranodal lymphoma with nonspecific clinical presentation. The coexistence of chylothorax and imaging findings suspicious for intratumoral hemorrhage in primary adrenal lymphoma is exceedingly rare and poorly understood. Herein, we report a case of bilateral primary adrenal diffuse large B-cell lymphoma (DLBCL) presenting with chylothorax secondary to lymphatic obstruction and imaging findings suspicious for intratumoral hemorrhage, highlighting the underlying pathophysiological mechanisms, multimodality imaging findings, and diagnostic challenges. A 67-year-old male patient presented with complaints of fever, night sweats, fatigue, and cough. Contrast-enhanced CT of the thorax and abdomen revealed pleural effusion in the left hemithorax. Thoracentesis was performed, and the patient was diagnosed with chylothorax. Concurrently, large bilateral adrenal masses with heterogeneous attenuation and focal hyperattenuating areas were identified. These hyperattenuating regions were considered suspicious for intratumoral hemorrhage. Subsequent PET/CT demonstrated intense hypermetabolic activity in both adrenal masses. An image-guided tru-cut biopsy was performed, and histopathological evaluation confirmed the diagnosis of diffuse large B-cell lymphoma. This case highlights that primary adrenal lymphoma may present with atypical manifestations such as chylothorax and imaging findings suspicious for intratumoral hemorrhage, emphasizing the importance of multimodality imaging, clinicopathological correlation, and consideration of this entity in the differential diagnosis of bilateral adrenal masses.

## Introduction

Primary adrenal lymphoma is a rare form of extranodal lymphoma, accounting for less than 1% of all cases of non-Hodgkin lymphoma.^1^^,^^2^ It typically occurs in the sixth decade of life and shows a male predominance. More than 70% of cases present with bilateral adrenal involvement.^3^^,^^4^ Histopathologically, the most common subtype is diffuse large B-cell lymphoma (DLBCL),^5^ which is characterized by an aggressive clinical course and rapid progression.

Clinical manifestations are generally nonspecific and often include systemic symptoms such as fever, night sweats, and weight loss. In addition, patients may present with localized symptoms such as abdominal or back pain. Features of adrenocortical insufficiency—including vomiting, skin hyperpigmentation, fatigue, and hypotension—may also be observed.^1^^,^^3^ Due to the aggressive nature of the tumor and its anatomical extent, atypical clinical presentations may occur.^6^

Vascular insufficiency secondary to the high proliferative rate of the tumor may predispose to intratumoral hemorrhage.^7^^,^^8^ Furthermore, chylothorax may develop as a result of mechanical compression of the retrocrural lymphatic network or the thoracic duct, even in the absence of direct malignant cell infiltration.^9^^,^^10^

A review of the literature indicates that the coexistence of chylothorax and adrenal-origin lymphoma is exceedingly rare. In this report, we aim to present a case of primary adrenal DLBCL with bilateral adrenal involvement, accompanied by chylothorax due to impaired lymphatic drainage in the left hemithorax and concurrent intratumoral hemorrhage, along with its clinical, radiological, and pathological findings.

## Case presentation

A 67-year-old male patient presented with complaints of fever, night sweats, cough, and fatigue. He had a 50 pack-year smoking history. His past medical history was significant for coronary artery disease requiring stent placement approximately 1.5 years earlier, as well as chronic obstructive pulmonary disease. There was no history of tuberculosis, known allergies, or alcohol use. On physical examination, decreased breath sounds were noted in the left basolateral lung field.

Laboratory investigations at admission revealed findings suggestive of increased tumor burden and systemic inflammation, including elevated serum lactate dehydrogenase (503 U/L), C-reactive protein (173 mg/L), ferritin (1211.4 µg/L), and erythrocyte sedimentation rate (65 mm/h). Among tumor markers, CA-125 was mildly elevated at 34.4 kU/L, while other markers were within normal limits. Complete blood count demonstrated mild anemia (hemoglobin: 12.7 g/dL), with no other significant abnormalities.

Contrast-enhanced thoracic CT performed at presentation demonstrated pleural effusion in the left hemithorax, reaching up to 4 cm in thickness. Diagnostic thoracentesis revealed chylous fluid, characterized by a triglyceride level >300 mg/dL and marked lymphocytic predominance, leading to the diagnosis of chylothorax. The patient was subsequently hospitalized for further evaluation.

**Figure 1:**
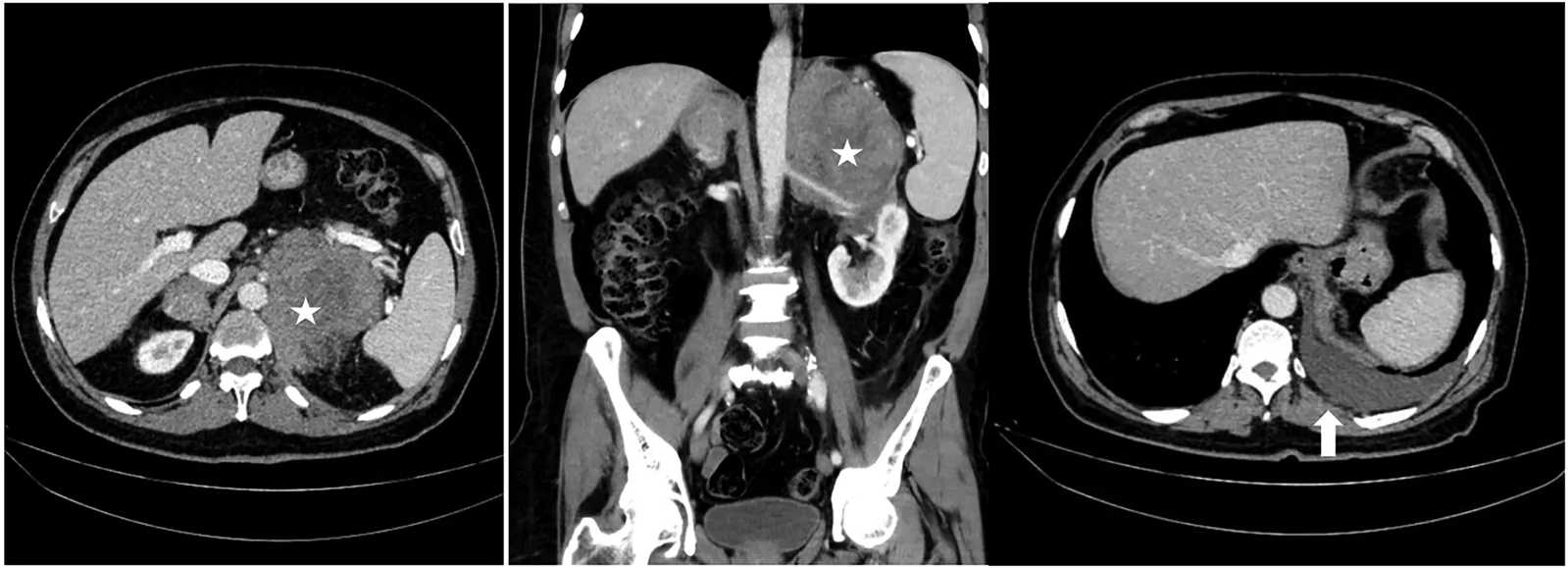
Contrast-enhanced abdominal CT axial and coronal images showing bilateral adrenal masses(asterisk) with hemorrhagic components at different stages. Axial contrast-enhanced chest CT scan showing left-sided pleural effusion (arrow) associated with chylothorax, confirmed by pleural fluid biochemical analysis.

Contrast-enhanced abdominal CT performed due to suspicion of malignancy demonstrated a large heterogeneous mass in the left adrenal gland measuring 110 × 88 × 126 mm, encasing and compressing the left renal artery and vein. The lesion contained focal hyperattenuating areas suspicious for intratumoral hemorrhage, although heterogeneous contrast enhancement could not be excluded in the absence of unenhanced CT images. A second heterogeneous lesion with similar imaging characteristics, measuring 45 × 40 × 64 mm, was identified in the right adrenal gland (Figure 1). Areas of low attenuation within the masses were interpreted as necrotic changes. No pathologically enlarged lymph nodes were detected in the retrocrural or paraaortic regions.

**Figure 2:**
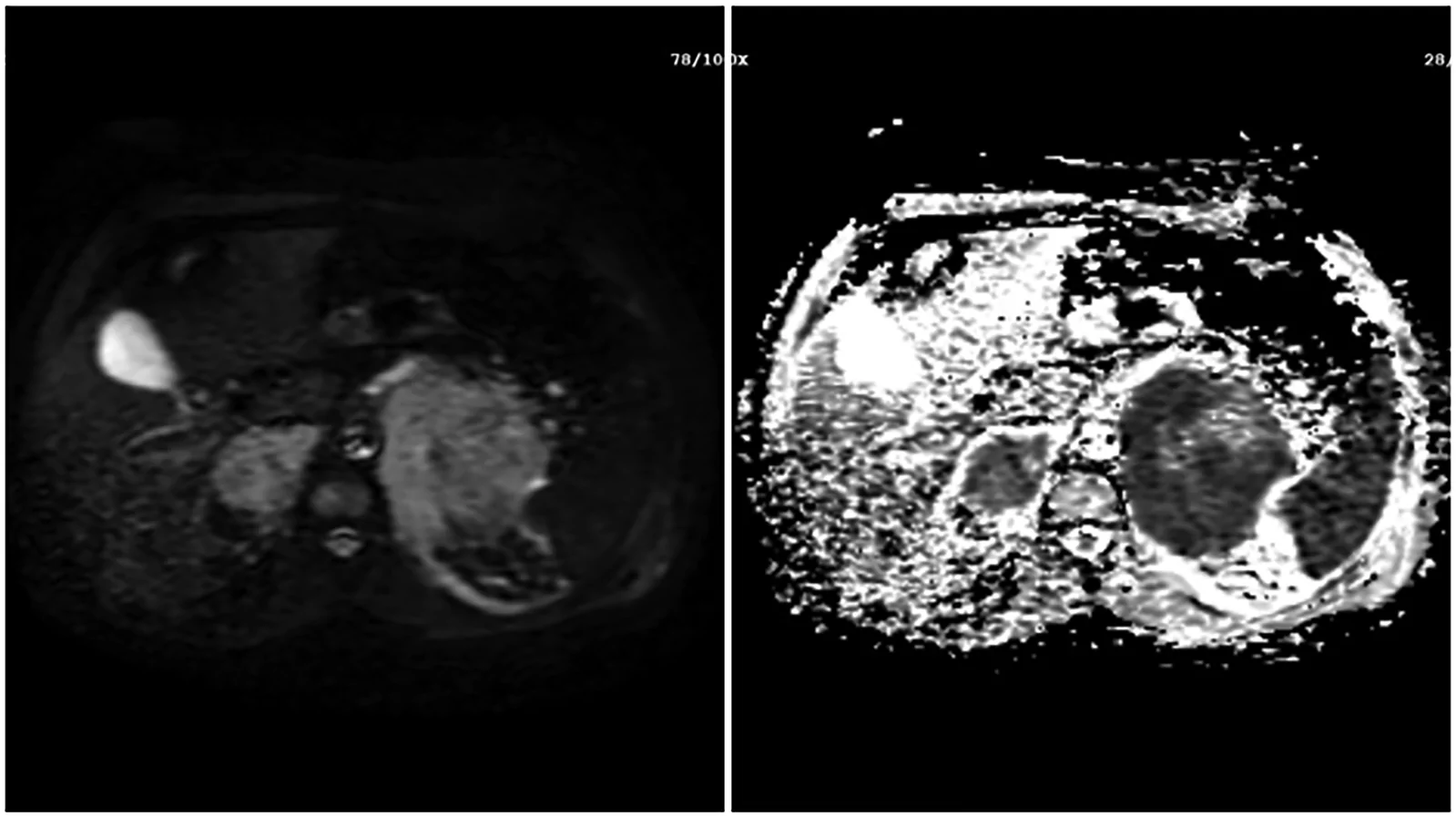
DWI/ADC images showing bilateral adrenal masses with marked diffusion restriction.

**Figure 3:**
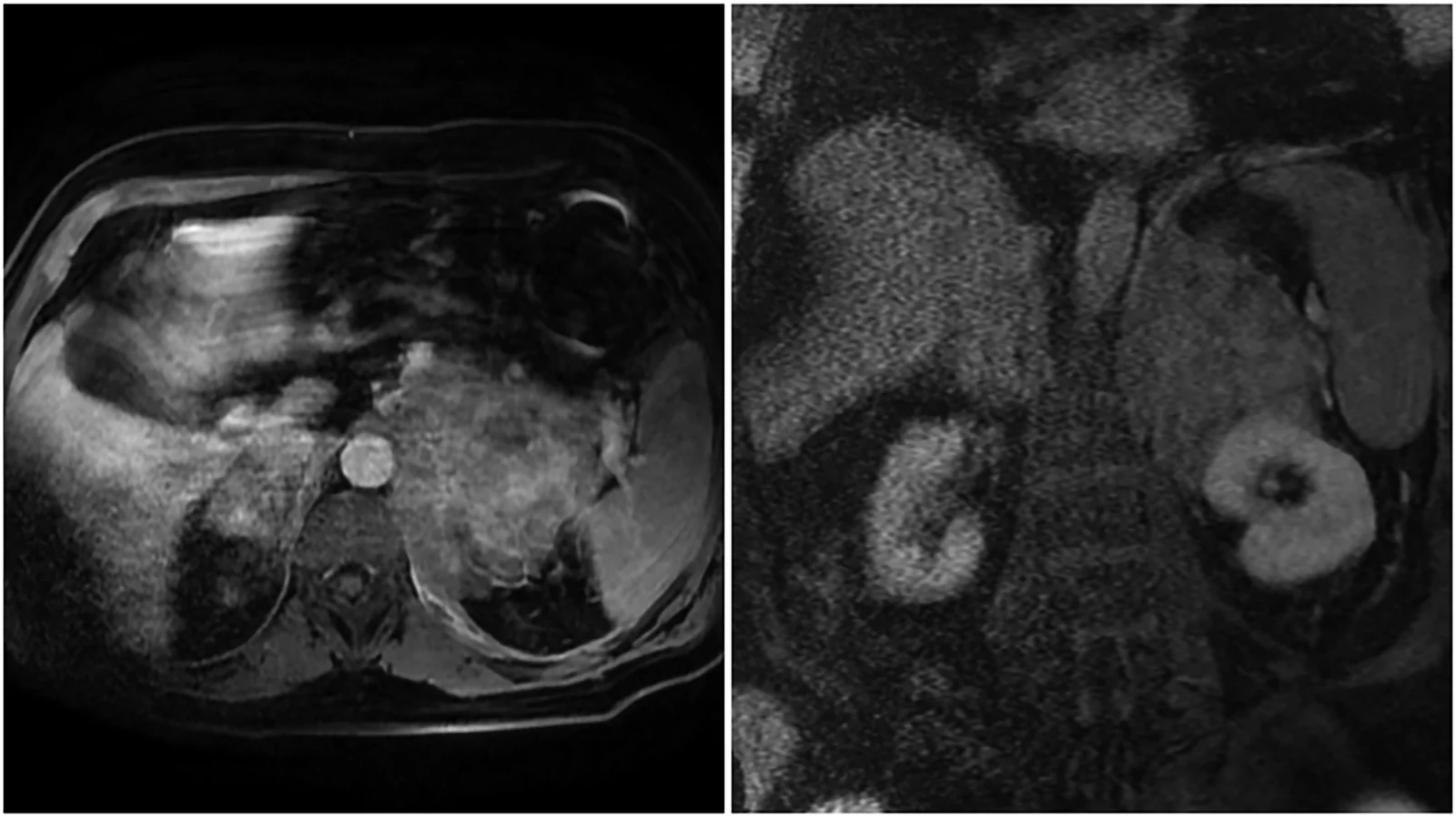
Post-contrast fat-suppressed T1-weighted axial and coronal MR images demonstrate bilateral adrenal masses with heterogeneous enhancement.

**Figure 4:**
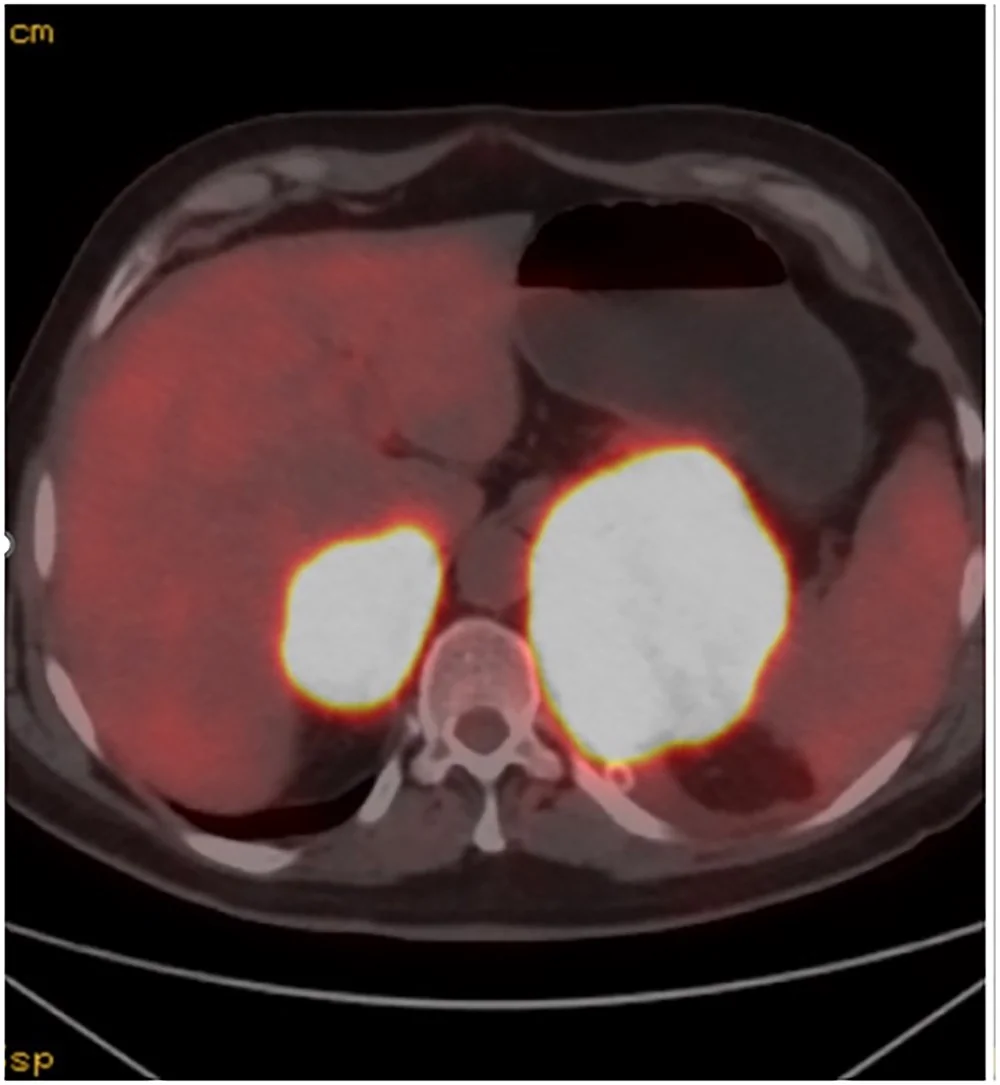
18F-FDG PET/CT fusion axial image showing increased FDG uptake in bilateral adrenal masses (SUVmax ∼ 30) and extension of the left adrenal mass into the paravertebral and retrocrural regions.

Subsequent MRI demonstrated diffusion restriction within the lesions (Figure 2) and heterogeneous contrast enhancement patterns (Figure 3). Due to persistent suspicion of malignancy, 18F-FDG PET/CT was performed, revealing a highly hypermetabolic mass in the left adrenal region measuring approximately 13 × 10 cm (SUVmax ≈ 30), extending into the paravertebral and retrocrural areas. A second lesion with similar metabolic characteristics, approximately 6 cm in size, was observed in the right adrenal gland (Figure 4). No pathological uptake was detected in the liver, spleen, or bone marrow. Additionally, no enlarged lymph nodes were identified in the abdomen or mediastinum.

An image-guided 18-gauge (18-G) tru-cut biopsy of the left adrenal mass was performed, and histopathological evaluation confirmed the diagnosis of DLBCL. Microscopic examination revealed diffuse infiltration with a non-germinal center B-cell phenotype. Immunohistochemical analysis showed positivity for CD20, BCL-2, BCL-6, and MUM1, along with a high proliferative index (Ki-67 approximately 80%). CD3, CD5, CD10, and c-MYC were negative. Bone marrow biopsy was normocellular, with no evidence of involvement. Furthermore, the absence of malignant cells in pleural fluid cytology suggested that the chylothorax was more likely due to mechanical compression of lymphatic structures rather than direct pleural lymphoma involvement.

Based on the overall clinical, radiological, and pathological findings, the patient was diagnosed with primary bilateral adrenal DLBCL. During hospitalization, serial follow-up revealed a rapid decline in hemoglobin levels. Although gastrointestinal bleeding was initially suspected, no active bleeding source was identified, the decrease was considered most likely secondary to suspected intratumoral hemorrhage. The patient received an erythrocyte suspension transfusion, resulting in hemodynamic stabilization and readiness for treatment.

Considering the patient’s cardiac history and the need to minimize cardiotoxicity, chemotherapy with the R-COEP regimen was initiated. The patient successfully completed the second cycle of R-COEP without complications and remains under outpatient follow-up.

## Discussion

Primary adrenal lymphomas are rapidly growing neoplasms that often reach large sizes at the time of diagnosis.^3^^,^^4^ In our patient, the left adrenal mass measured up to 13 cm, with a maximum standardized uptake value (SUVmax) exceeding 30 on PET/CT, reflecting the aggressive biological behavior of the tumor, which is also supported by a high Ki-67 proliferation index of approximately 80%.^5^^,^^6^

In lymphomas, insufficient tumor neovascularization relative to the rapid proliferative rate (Ki-67: 80%), together with the rich arterial supply of the adrenal glands, may predispose to ischemic necrosis and subsequent hemorrhage.^7^^,^^8^ In our case, contrast-enhanced CT demonstrated focal hyperattenuating areas within the bilateral adrenal masses. Although these findings, together with the rapid decline in hemoglobin levels during hospitalization, raised suspicion for spontaneous intratumoral hemorrhage, unenhanced CT was not available, and heterogeneous contrast enhancement cannot be completely excluded. Bilateral adrenal masses also demonstrated hypoattenuating areas, likely representing necrotic change.

In the differential diagnosis, adrenocortical carcinoma, metastatic lesions, pheochromocytoma, and infectious etiologies should be considered. However, bilateral involvement together with intense FDG uptake and the overall imaging appearance should prompt consideration of lymphoma in the differential diagnosis.^2^

Perhaps the most striking feature of this case is the etiology of the rare complication of chylothorax. Malignancy-associated chylothorax is typically attributed to direct invasion of the thoracic duct or compression by mediastinal lymphadenopathy.^9^^,^^10^ In this case, however, the absence of malignant cells in pleural fluid cytology supports a mechanical rather than infiltrative cause. PET/CT imaging demonstrated that the mass extended into the paravertebral region and retrocrural space at the T11-T12 level. This anatomical extension likely resulted in external compression of the cisterna chyli or the proximal thoracic duct at the level of the diaphragmatic crura, leading to impaired lymphatic drainage and subsequent development of chylothorax.

A review of the literature indicates that chylothorax is more commonly associated with mediastinal lymphomas, and cases caused by retroperitoneal primary adrenal lymphoma are exceedingly rare.^1^^,^^10^ Furthermore, the coexistence of chylothorax and bilateral primary adrenal lymphoma is exceptionally rare. This case highlights the diagnostic value of multimodality imaging together with clinicopathological correlation in establishing the diagnosis.

The most notable contribution of this case to the literature is the proposed mechanism of chylothorax secondary to retroperitoneal lymphatic obstruction, supported by multimodality imaging and clinicopathological findings.

## Conclusion

Primary bilateral adrenal lymphoma is a rare but highly aggressive malignancy that may present with atypical manifestations such as chylothorax and imaging findings suspicious for intratumoral hemorrhage. Radiologically, large bilateral adrenal masses with heterogeneous attenuation, hyperattenuating hemorrhagic components, diffusion restriction, and intense FDG uptake should raise strong suspicion for lymphoma.

In patients with unexplained chylous pleural effusion, radiologists should consider secondary lymphatic obstruction due to retroperitoneal or adrenal masses. Early recognition and image-guided biopsy are essential for prompt diagnosis and initiation of appropriate therapy.

## Learning points

- Primary adrenal lymphoma is a rare but aggressive entity that typically presents with bilateral adrenal involvement and intense FDG uptake on imaging.
- In patients with unexplained chylous pleural effusion, secondary lymphatic obstruction due to retroperitoneal or adrenal masses should be considered, as chylothorax may result from mechanical compression rather than direct tumor infiltration.
- Early recognition of characteristic imaging findings and prompt image-guided biopsy are essential for accurate diagnosis and timely management.
